# Effects of vitamin D supplementation on depression and some selected pro-inflammatory biomarkers: a double-blind randomized clinical trial

**DOI:** 10.1186/s12888-022-04305-3

**Published:** 2022-11-11

**Authors:** Mina Kaviani, Bahareh Nikooyeh, Farnaz Etesam, Siroos Jahangiri Behnagh, Hamed Mohammadi Kangarani, Mohammad Arefi, Parichehreh Yaghmaei, Tirang R. Neyestani

**Affiliations:** 1 Department of Science Translation and Public Food and Nutrition Education, Faculty of Nutrition and Food Technology, National Nutrition and Food Technology Research Institute, No.7- Shahid Hafezi (West Arghavan) St., Farahzadi Blvd., Sanat Sq. Shahrak Qods (Gharb), Tehran, 1981619573 Iran; 2 Laboratory of Nutrition Research, Faculty of Nutrition and Food Technology, National Nutrition and Food Technology Research Institute, No.7- Shahid Hafezi (West Arghavan) St., Farahzadi Blvd, Sanat Sq. Shahrak Qods (Gharb), Tehran, 1981619573 Iran; 3 Imam Khomeini Hospital Complex, Tohid Sq, Tehran, 1419733141 Iran; 4 Jahan Hekmat clinic, No. 1- East Aseman 4, Paknejad Blvd, Tehran, Saadat abad Iran; 5 Railway Sq, Baharloo Hospital, Behdari St, Tehran, Iran; 6 Department of Biology, Faculty of Basic Sciences, Science and Research Branch, Islamic Azad University, Daneshgah Blvd, Simon Bulivar Blvd, Tehran, 1477893855 Iran

**Keywords:** Depression, Vitamin D, Pro-inflammatory biomarkers, RCT

## Abstract

**Background:**

Both augmented inflammatory reaction and low vitamin D status are associated with depression but the magnitude of their relationships is unclear. This study was, therefore, conducted to evaluate the effects of vitamin D supplementation on serum 25(OH)D concentration, depression severity and some pro-inflammatory biomarkers in patients with mild to moderate depression.

**Methods:**

An 8-week double-blind randomized clinical trial (RCT) was performed on 56 (18–60 yrs) patients with mild to moderate depression, randomly assigned to intervention (50,000 IU cholecalciferol 2wks^−1^) and control (placebo) groups. Serum 25(OH)D, intact parathyroid hormone (iPTH), interlukin (IL)-1β, IL-6, high-sensitivity C-reactive protein (hs-CRP) and depression severity (Beck Depression Inventory-II) (BDI-II)) were initially and finally assessed.

**Results:**

At the end point, statistically significant changes were observed only in intervention group as compared with controls including increased 25(OH)D concentration (+ 40.83 ± 28.57 vs. + 5.14 ± 23.44 nmol L^−1^, *P* < 0.001) and decreased depression severity (-11.75 ± 6.40 vs. -3.61 ± 10.40, *P* = 0.003). No significant within- or between group differences were observed in serum IL-1β, IL-6 and hs-CRP concentrations.

**Conclusion:**

Increased circulating 25(OH)D concentrations following 8-week vitamin D supplementation (50,000 IU 2wks^−1^) resulted in a significant decrease in BDI-II scores in patients with mild to moderate depression. However, this effect was independent of the serum concentrations of the studied inflammatory biomarkers.

**Trial registration:**

The clinical trial registration code was obtained from the Iranian Registry of Clinical Trials (date of registration: 17/09/2018, registration number: IRCT20170926036425N1) and ClinicalTrials.gov (date of registration: 04/12/2018, registration number: NCT03766074)

## Introduction

### Background and objectives

Depression has been, and continues to be, a serious threat to the people’s mental health with an accelerated occurrence rate due to the newly emerged coronavirus disease (COVID-19) pandemic [1]. Due to the worldwide condition of home-quarantine, economic problems [1, 2] and side effects of antidepressants [3], finding simple effective practical strategies to control depression seems necessary [4]. Hence, the importance of finding the relationship between factors involved in depression is self-evident [5]. Depression is proposed the result of disorders in inter-neurons signaling that could cause difficulties in connection of different parts of brain via imbalance of stimulating and inhibitory neurons interactions [6]. Recently, an interesting theory suggests that increasing in intracellular calcium^+2^ (Ca^+2^) is one of main causes of depression at cellular level [6]. Despite progression in discovering mechanisms involved in depression such as inflammation, hypothalamic–pituitary–adrenal (HPA) axis and vitamin D [7, 8], the magnitude of their relationships is still unclear. In this context, the importance of inflammation is such that some researchers consider it as the main cause of depression [9]. It is believed that even psychosocial factors and bitter events of life, first cause an increase in pro-inflammatory biomarkers such as IL-1β, IL-6 and hs-CRP which is then followed by depression [10–12]. Study on people exposed to chronic inflammation has shown a two-way relationship between inflammation and depression [6]. This means that depression due to changes in function of parts of brain that are in relation with mood including hippocampus and hypothalamus could increase pro-inflammatory cytokines [6]. On the other hand, it has been suggested that increasing of pro-inflammatory cytokines including IL-1β, IL-6 and hs-CRP elevates function of mitochondria and reactive oxygen species (ROS) that by affecting function of neurons could cause depression [6].

IL-1β with T cell-stimulating ability plays a role in structure and function of neurons and their immunity responses [13]. Recently, it has been shown that IL-1β is associated with depression [6, 14] but the magnitude of this association and its relationship with other involved factors in depression needs more clarification.

IL-6, a pleiotropic cytokine, plays regulatory roles in immunity responses, acute phase reactions and in differentiation of some neurons, as well [13, 14]. As an inflammatory mediator, upregulation of IL-6 has been associated with several human diseases including obesity, inflammatory bowel disease, certain types of malignancies and ocular inflammation [15–18]. Some studies have suggested a role for IL-6 in development of depression. Even an experimental study reported resistance to stress-induced depression behavior in IL-6 knockout mice [19]. Nevertheless, the underlying mechanisms of IL-6 function and associations with other possible involved factors in depression are still unknown.

The association of C-reactive protein (CRP), another inflammatory biomarker, with depression has also been investigated. Recent studies reported elevated serum CRP concentrations in the subjects with major depressive disorder (MDD) [20, 21] and especially in those who were resistant to treatment [20]. Though the augmented inflammatory reaction in MDD might be due to smoking and dietary habits, the association between CRP and MDD remained significant even after complete adjustment for all confounders [21]. It is still unknown whether the association between inflammation and depression has a pathophysiological basis [20, 21] or it is due to some overlooked psychosocial and/or clinical confounders [21].

Regarding the repeatedly documented association of diet and inflammation [22], dietary approaches have been considered to ameliorate depression through targeting specific pathways involved in inflammation including oxidative stress, HPA and obesity, among the others [23].

One of the most studied dietary factors in relation to mental disorders is vitamin D. Exploration of vitamin D receptor (VDR) in the central nervous system (CNS) opened new insights into the so-called “non-calcemic” functions of this secosteroid. New studies indicate a wide spectrum of activities for vitamin D in CNS including energy hemostasis through central renin-angiotensin system (RAS) [24], protective effect against autoimmunity through attenuation of micoglia activation via neuron-specific signaling [25] and overall mental health in both children and adults [26, 27]. The anti-inflammatory and anti-oxidant properties of vitamin D have already been documented in other clinical settings [28–31], but assessment of these effects in relation to depression needs more studies. We have recently reported the effect of vitamin D supplementation on depression and certain neurotransmitters [32]. In this piece of work, we examined the associations among vitamin D status, depression and inflammation in an RCT.

## Methods

### Study design

This eight-week double-blind RCT was conducted on subjects with mild to moderate depression referred to the outpatient clinics of Baharloo Hospital, between May 2018 and June 2019, Tehran, Iran. The participants had no other psychiatric disease. The detailed protocol of this study can be found elsewhere [33].

A sample size of 56 subjects was calculated through considering an effect size of 0.75 and a power of 80% based on previous study [34] according to the following formula, with 28 patients randomly assigned to each group following simple randomization procedures (according to entrance code) by head of the project [33].

$$n=\left[\frac{{2\left({Z}_{1-a/2}+{Z}_{\beta }\right)}^{2}{SD}^{2}}{{\left({\mu }_{1}-{\mu }_{0}\right)}^{2}}\right]$$

The inclusion criteria were [1] 18–60 years of age and [2] having mild to moderate depression with no other psychiatric disease, as confirmed by the psychiatrist. We enrolled both incident and old cases of depression.

The non-inclusion criteria were: [1] having a history of heart infarction, angina pectoris, stroke, kidney stones, high blood pressure, liver disease, and hyperparathyroidism, [2] pregnancy and/or lactation, [3] reproductive-aged women (under 50 years old) not receiving adequate contraception, [4] consuming nutritional supplements containing vitamin D from two months prior to the intervention. Exclusion criteria were: [1] lack of willingness to continue the study and [2] failure to follow the interventional program. More details were described in protocol of the study [33].

After describing the protocol and objectives of the study to the registered volunteers, further evaluations were done on enrolled participants by psychiatrists including determination of depression severity using BDI-II questionnaire at the baseline and after the intervention. Following obtaining written informed consent, the participants were randomly allocated to either intervention or control group and received either 50,000 IU cholecalciferol 2wks^−1^ or placebo, respectively. Vitamin D_3_ supplements and placebos (made from oral paraffin) were purchased from Zahravi Pharmaceutical Company (Iran). Safety considerations to select a safe dose of vitamin D supplements were based on upper tolerable intake level of vitamin D for adults (4,000 IU day^−1^) [35], and the results pertaining to previous studies with higher doses of vitamin D (50,000 IU wk^−1^) [36]. All participants had three visits (weeks 0, 4 and 8). On the first visit, general socio-demographic data including medical history, exposure to sunlight, sunscreen use, tobacco and drug use habits and alcohol consumption were obtained during a face to face interview and completing a questionnaire. The physical activity level (PAL) was assessed using a modified questionnaire of “Intensity and Effects of Various Activities on Physical Activity level in Adults” [35]. Blood pressure and anthropometric measures were also taken. All assessments were performed at the beginning and end of the study. Subjects were instructed to stick to their usual diet, PAL, and medications during the intervention period, as prescribed by their physician.

**Fig. 1:**
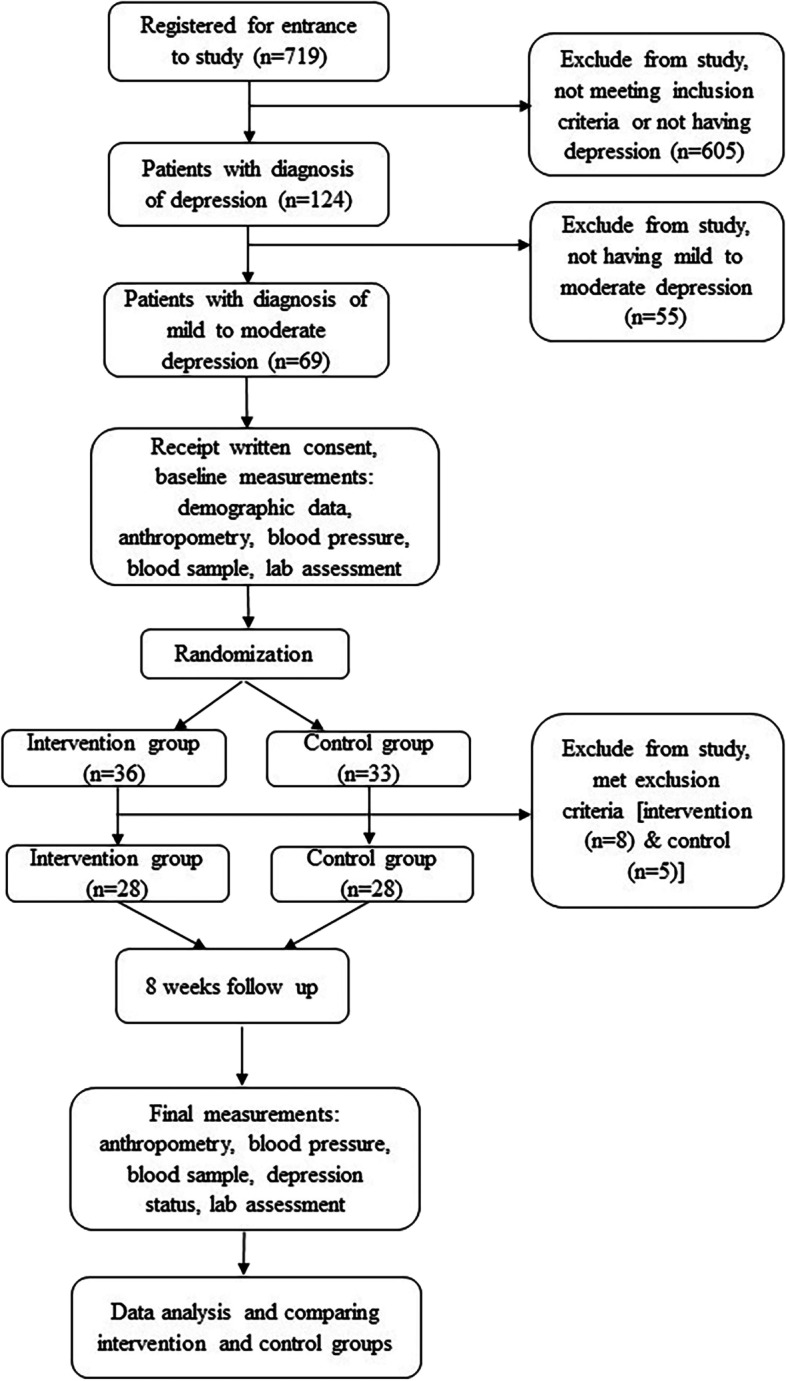
Summery of the study design

Subjects were received their pills (vitamin D_3_ or placebo) in the first and second visits. Supplements and placebos were completely similar in appearance and packaging. None of the researchers or patients were aware of the treatment assignments except head of the project. Blinding in current study was done by writing the entrance code of subjects on the package of pills. All participants received a reminder call and a written "instruction of use" to consume the remaining pills. They were also asked to return the pills that were not consumed for any reason in their next visit. Therefore, the adherence assessment was carried out based on pill count and patient self-report. Summary of the study design has been shown in Fig. 1.

### Assessment of depression

Assessment of depression was carried out according to the psychiatrist's assessments through the structural clinical diagnostic interview based on Diagnostic and Statistical Manual of Mental Disorders, Fourth Edition (DSM–IV) criteria and BDI-II score. It is noteworthy that accuracy and reliability of BDI-II questionnaire [37], as well as, translation, cultural adaptation, and validation of its Persian version [38] have been proven before. In this study, mild to moderate depression was defined as a BDI-II score ranging from 13 to 29 [37].

### Anthropometric measures

Anthropometric assessments including weight, height, waist circumference (WC) and hip circumference (HC) were done. Body mass index (BMI) and waist to hip ratio (WHR) were calculated by dividing weight (kg) by height^2^ (m^2^) and WHR by dividing WC to HC, respectively. More details were described elsewhere [38].

### Blood pressure

Systolic and diastolic blood pressures (SBP and DBP) were measured in sitting position after 10 min resting using digital sphygmomanometer (BC 08; Beurer, Ulm, Germany).

### Laboratory investigations

Briefly, a 10-ml venous blood sample was collected from all participants and stored on ice in cold boxes for transportation to the Laboratory of Nutrition Research, National Nutrition and Food Technology Research Institute (NNFTRI). Sera were separated from the clot samples by centrifugation at room temperature and then aliquoted in fresh microtubes and stored at -80 °C until use. In this study, enzyme immunoassay (EIA) method was employed for assessing all biochemical parameters as follows: serum 25(OH)D (Euroimmun EIA kit, Lubeck, Germany), iPTH (Euroimmun EIA kit, Lubeck, Germany), hs-CRP (Zellbio EIA kit, Ulm, Germany), IL-1β (Diaclone EIA kit, Besancon, France) and IL-6 (IBL EIA kit, Hamburg, Germany). Details of laboratory methods were described previously [33].

### Classification of vitamin D status

Vitamin D status was classified based on circulating concentration of 25(OH)D as deficiency (< 50 nmol l^−1^), insufficiency (50–75 nmol l^−1^) and sufficiency (> 75 nmol l^−1^) [39].

### Outcomes

The primary outcome was the significant elevation of serum 25(OH)D concentration from baseline until the end of intervention. Secondary outcomes included significant changes in serum pro-inflammatory biomarkers including IL-1β, IL-6 and hs-CRP. Other secondary outcomes were significant changes in serum iPTH and depression severity (BDI-II score) from baseline to the end of the eight-week intervention. More details were described elsewhere [33].

### Statistical analyses

Quantitative and qualitative data were expressed as mean ± standard deviation (SD) and absolute or relative frequencies, respectively. The normality of data distribution was assessed by Shapiro–Wilk's test. To compare qualitative variables between the groups at baseline Chi-square test was used. Based on the study design, two groups were investigated within two time periods (before and after intervention); thus, paired-sample *t*-test or Wilcoxon test (based on the normal or non-normal distribution of data, respectively) was utilized to compare within-group changes and independent sample *t*-test or Mann–Whitney test (based on the normal or non-normal distribution of data) was used to compare between-group changes. The significance level was *P* < 0.05. Data were analyzed using Statistical Package for Social Sciences (SPSS) software v.21 (SPSS Inc., Chicago, IL, USA). Analysis was done by intention to treat. Further details were previously described [32].

### Ethics

Ethical approval was obtained from the Ethics Committee of NNFTRI (IR.SBMU.NNFTRI.REC.1396.185) and all methods were performed in accordance with the relevant guidelines and regulations (NNFTRI). The clinical trial registration code was obtained from the Iranian Registry of Clinical Trials (IRCT20170926036425N1) and ClinicalTrials.gov (NCT03766074). Written informed consent was obtained from all participants or their next of kin/legally authorized representative (according to educational level) before entering the research, as well.

## Results

Out of the 719 registered volunteers, 69 participants (56 incident and 13 old cases) were enrolled in the study, with almost equal number of old cases in each group (6 in intervention and 7 in control group). However, 13 subjects were excluded due to meeting exclusion criteria. Consequently, 56 subjects (intervention *n* = 28, control *n* = 28) completed the study. Participants were 50 women (89.29%) and 6 men (10.71%) aged 43.0 ± 1.15 yrs. Estimated adherence of the participants to the study protocol was approximately 100%. There was no complain about adverse drug reactions and no report of suicide attempt.

General characteristics and other parameters of the study groups had no statistically significant differences at the baseline (Tables 1 and 2). Furthermore, there were no significant within- and between-group differences in the final values of anthropometric indices, SBP, and DBP.

**Table 1:** General characteristics of participants at base line

Variable		Study group		P^*^ value
		Intervention (*n* = 28)	Control (*n* = 28)	
Sex^a^	Female Male	27 (96.4) 1 (3.6)	23 (82.1) 5 (17.9)	0.193
Age (year)^b^		43.14 (9.25)	42.86 (8.01)	0.902
Educational level^a^	Illiterate & elementary Guidance & high school Diploma University	7 (25) 6 (21.4)	3 (10.7) 6 (21.4)	0.50
		7 (25) 8 (28.6)	7 (25) 12 (42.9)	
marital situation^a^	ingle Married Divorced	2 (7.1) 26 (92.9) -	3 (10.7) 24 (85.7) 1 (3.6)	0.53
Sunlight exposure a day^a^	No exposure 10–60 min > 60 min	8 (28.6) 18 (64.3) 2 (7.1)	5 (17.9) 22 (78.6) 1 (3.5)	0.49
Time of sunlight exposure^a^	10 AM- 15 PM Other times	18 (64.3) 10 (35.7)	12 (44.4) 15 (55.6)	0.14
Duration of Sunlight exposure^b^ (min)		26.61 (25.09)	24 (16.76)	0.86
Sunlight exposure ^a^ (part of body)	Face feet Hand from wrist Hand from arm Combination of above	8 (28.6) 1 (3.6)—1 (3.6) 18 (64.3)	3 (10.7)—2 (7.1) 1 (3.6) 22 (78.6)	0.23
Sunscreen usage^a^	Never Occasionally Often always	13 (46.4) 5 (17.9) 2 (7.1) 8 (28.6)	13 (46.4) 8 (28.6) 2 (7.1) 5 (17.9)	0.71
Drug usage^a^	Never Very low Low Moderate High	23 (82.1) 3 (10.7) 1 (3.6) 1 (3.6)	23 (82.1) 3 (10.7) 1 (3.6) 1 (3.6) -	0.74
Alcohol consumption^a^	Never Very low Low	26 (92.9) 1 (3.6) 1 (3.6)	21 (75) 6 (21.4) 1 (3.6)	0.13
Physical activity level ^a^	Very low Low Moderate High Very high	17 (60.7) 9 (32.1) 2 (7.1) - -	15 (53.6) 8 (28.6) 3 (10.7) 1 (3.6) 1 (3.6)	0.67
vitamin D status of study groups^c a^	Deficiency insufficiency sufficiency	1(3.6) 9 (32.1) 18 (64.3)	7 (25) 9 (32.1) 12 (42.9)	0.058
Anthropometric measurements	Weight	75.72 (12.22)	75.15 (16.73)	0.88
	BMI [kg (m2)-1]	29.98 (4.64)	28.55 (5.33)	0.29
	Waist circumference (cm)	98.91(10.54)	97.75 (12.27)	0.71
	Hip circumference (cm)	113.39 (8.29)	112.32 (11.70)	0.69
	WHR	0.87 (0.07)	0.87 (0.07)	0.84
Blood pressure	SBP (mm Hg)	122.25 (14.11)	120.18 (13.06)	0.57
	DBP (mmHg)	79.79 (10.78)	77.75 (10.31)	0.52

*BMI* Body mass index, *WHR* Waist to hip ratio, *SBP* Systolic blood pressure, *DBP* Diastolic blood pressure

^a^Number (%)

^b^Mean (± SD)

^c^Vitamin D status was classified based on circulating concentration of 25(OH)D as deficiency (< 50 nmol l^−1^), insufficiency (50–75 nmol l^−1^) and normal status (> 75 nmol l^−1^)

^*^Denotes the significance of differences between the study groups, chi-square test for qualitative data and for quantitative data, independent sample *t*-test (for normal distribution), Mann–Whitney test (for non-normal distribution)

**Table 2:** Baseline characteristics and comparison of the changes within and between groups following the intervention^a^

Variable	Group							
	Intervention (*n* = 28)			Control (*n* = 28)			Between-group P** value	
	Before	After	P^*^ value	Before	After	P^*^ value	Before	After
25(OH)D (nmol L^−1^)	87.1 (28.55)	127.92 (24.93)	< 0.001	73.64 (31.94)	78.77 (27.05)	0.26	0.10	< 0.001
Serum iPTH (pg mL^−1^)	13.26 (14.39)	13.43 (9.30)	0.07	11.08 (8.33)	16.18 (7.92)	< 0.001	0.64	0.07
hs-CRP (mg L^−1^)	4.40 (3.03)	4.67 (3.39)	0.7	5.29 (4.57)	4.07 (3.71)	0.13	0.97	0.18
IL-1β (pg mL^−1^)	39.83 (33.15)	41.04 (36.45)	0.67	30.85 (34.15)	33.76 (28.80)	0.41	0.15	0.76
IL-6 pg mL^−1^)	2.08 (0.63)	1.98 (0.44)	0.85	3.23 (5.27)	3.39 (6.66)	0.13	0.26	0.31
BDI-II score	23.86 (5.49)	12.11 (6.12)	< 0.001	21.79 (5.74)	18.18 (12.82)	0.053	0.23	0.003

*25(OH)D* 25-hydroxyvitamin D, *iPTH* intact parathormone, *hs-CRP* high-sensitivity C-reactive protein, *IL-1β* interleukin-1β, *IL-6* interleukin-6, *BDI-II* Beck Depression Inventory-II

^a^All values are means (± SDs)

^*^Denotes the significance of within-group changes, paired sample *t*-test (for normal distribution), Wilcoxon test (for non-normal distribution)

^**^Denotes the significance of between-group changes, independent sample *t*-test (for normal distribution), Mann–Whitney test (for non-normal distribution)

Following the intervention, the rise in 25(OH)D concentration was significantly higher in the intervention group compared with the control group (+ 40.83 ± 28.57 vs. + 5.14 ± 23.44 nmol L^−1^, *P* < 0.001) (Table 2). Vitamin D status of both study groups had no significant difference at the beginning of the study (Table 1); however, after intervention it was significantly improved only in the intervention group compared with control one (0 (0), 1 (3.6), 27 (96.4) vs. 4 (14.3), 7 (25), 17 (60.7) as deficiency, insufficiency and sufficiency, respectively, n (%), *P* = 0.005)).

Final serum iPTH concentrations of both study groups had no statistical difference. However, there was a significant within-group increase just in the control group (+ 5.10 ± 6.002 pg mL^−1^, *P* < 0.001) (Table 2).

After intervention a significant reduction was observed in BDI-II scores of intervention group compared to baseline (-11.75 ± 6.40, P < 0.001) and this reduction was significant compared to the control group, as well (-11.75 ± 6.40 vs. -3.61 ± 10.40, *P* = 0.003); however, reduction of the BDI-II score of control group showed no significant changes compared to baseline (Table 2).

There was no significant within- or between-group difference of the initial and final serum concentrations of IL-1β, IL-6 and hs-CRP (Table 2).

## Discussion

We found that expectedly increased circulating 25(OH)D concentrations following eight weeks supplementation in patients with mild to moderate depression was accompanied by a significant recuperation of depression severity as compared with the control group. However, this intervention did not significantly affect the inflammatory status, as judged by serum IL-1β, IL-6 and hs-CRP concentrations.

Similar to the present study, previous studies have reported significant improvement in depression severity following vitamin D supplementation [36, 40–46]. Recently some regulating roles on mood were attributed to vitamin D due to production of its active form in brain and also gene expression of VDRs especially in areas in relation with mood and social behaviors [6, 47–52]. Considering recent theory about increasing intra-neuron Ca^+2^ concentration as a responsible factor for disturbing balance between inhibitory and stimulating neurons [6], it has been suggested that vitamin D may have regulating roles on intra-neuron Ca^+2^ concentrations due to its gene-dependent function [50, 53, 54]. Vitamin D is likely to decrease Ca^+2^ signaling through several mechanisms including: (i) up-regulation of calbandin and parvalbumin genes expression whereby converting Ca^+2^ to its buffer forms [9]; (ii) up-regulation of Ca^+2^ pump (PMCA) and Na^+^/Ca^+2^ exchanger (NCX1) in plasma membrane and hence egression of extra Ca^+2^; and (iii) down-regulation of L-type Ca^+2^ channels (CaV1.3 & CaV1.2) of neurons of hippocampus and cortex [9]. However, some studies failed to show significant improvement in depression severity after vitamin D supplementation [34, 55–57]. These controversies may be attributed to design of the studies, initial serum 25(OH)D concentrations of the participants, dose and type of vitamin D supplement (D_2_ vs. D_3_), intervention duration, method of supplementation, age of the target group, existence of other comorbidities and additional simultaneous interventions. Among these, baseline circulating concentrations of 25(OH)D as well as dose and type of vitamin D supplement are of particular importance [55]. It is noteworthy that most participants of the present study had optimal levels of serum 25(OH)D at the baseline. Nevertheless, vitamin D supplementation was still efficacious in lessening depression severity. It is likely that “more than adequate” vitamin D should be targeted in patients with depression.

In the current study, eight weeks supplementation with vitamin D was not able to significantly change serum concentrations of pro-inflammatory biomarkers including IL-1β, IL-6 and hs-CRP in the subjects with mild to moderate depression. The results of some previous observational studies showed an inverse association between circulating concentrations of inflammatory mediators (IL-6 and hs-CRP) and 25(OH)D in subjects with depression [8, 58]. However, the results of RCTs aimed to investigate the effects of vitamin D on depression severity and pro-inflammatory biomarkers have been controversial. In agreement with our results, an interventional study showed that supplementation with vitamin D (50,000 IU 2wks^−1^) for eight weeks did not decrease serum IL-6 [59] and hs-CRP concentrations in adult subjects with depression [60]. In contrast, some investigations reported a significant suppressive effect of 12 weeks vitamin D supplementation (50,000 IU 2wks^−1^) on serum hs-CRP in depressed patients as compared with control group [43, 61].

Since inflammation can affect the neuronal functions responsible for depression in several ways, anti-inflammatory factors such as vitamin D may play a role in controlling inflammation and hence depression severity through various mechanisms [9]. It should be noted that inflammation elevates the function of mitochondria and production of ROS that deeply affect the function of neurons [9]. It seems ROS by inhibiting the synthesis of key neurotransmitters involved in depression such as serotonin and also increasing the Ca^+2^ signaling through increasing the sensitivity of inositol triphosphate (IP3) receptors and depleting of glutathione resources of neurons can contribute in development of depression [9]. Following finding of VDRs in brain, it was suggested that gene-dependent function of vitamin D probably could decrease the production of ROS by affecting gene expression of DNA^1^-demethylase through inhibition of hyper-methylation of promoters responsible for gene transcription [9]. On the other hand, it was proposed that vitamin D may also control the function of ROS probably by maintenance of the homeostasis of serotonin through modulation of gene expression of tryptophan hydroxylase 1 & 2 (TPH1 & TPH2), the key enzymes of tryptophan synthesis pathways. Besides, vitamin D may also affect the function of ROS by up-regulation of glutathione to compensate for lost resources [9].

Interestingly, most commonly used anti-depressant medications, including Fluxetine, have anti-inflammatory properties [62, 63] and even recently have been proposed as an anti-inflammatory therapy against newly emerged coronavirus infection [64].

### Limitations

This study had some limitations. Firstly, short-term effects of vitamin D supplementation on severity of depression may not necessarily reflect the long-term outcomes. Secondly, most of our participants had adequate baseline circulating 25(OH)D concentrations, which may have concealed any possible association among depression, inflammation and vitamin D. There is a great need for well-designed RCTs with longer durations to elucidate the possible effects of vitamin D on depressive disorders. Besides, simultaneous evaluation of vitamin D supplementation effects on glutathione and cytokines in depressed patients could be very helpful to explore the novel pathways of depression pathogenesis. Additionally, evaluation of the effects of vitamin D on gene expression of PMCA, NCX1 and L-type CaV1.2 and CaV1.3 necessitates further research. Current study had some advantages including, double-blind RCT design, safe dose of vitamin D supplements and control of confounding factors (e.g., anthropometric indices, SBP and DBP).

## Conclusion

Eight-week supplementation with vitamin D (50,000 IU 2wks^−1^) resulted in a significant increase in serum 25(OH)D concentrations of adult subjects with mild to moderate depression which was accompanied with amelioration for their depression severity. This effect was independent of circulating concentrations of IL-1β, IL-6 and hs-CRP.

Our findings shed some light on the mechanisms underlying the pathophysiology of depression and may also be helpful for future preventive and therapeutic approaches.

## Data Availability

The data used and analyzed during the current study are available from the corresponding author.

## Abbreviations

BDI-II: Beck Depression Inventory-II
BMI: Body mass index
COVID-19: Coronavirus disease
CRP: C-reactive protein
Ca^+2^: Calcium^+2^
CNS: Central nervous system
DBP: Diastolic blood pressure
DSM–IV: Diagnostic and Statistical Manual of Mental Disorders, Fourth Edition
EIA: Enzyme immunoassay
HC: Hip circumference
HPA: Hypothalamus–pituitary–adrenal
hs-CRP: High-sensitivity C-reactive protein
IL: Interlukin
iPTH: Intact parathyroid hormone
IP3: Inositol triphosphate
CaV1.3 & CaV1.2: L-type Ca^+2^ channels
MDD: Major depressive disorder
NCX1: Na^+^/Ca^2+^ exchanger
NNFTRI: National Nutrition and Food Technology Research Institute
PAL: Physical activity level
PMCA: Calcium pump
RCT: Randomized clinical trial
RAS: Renin-angiotensin system
ROS: Reactive oxygen species
SD: Standard deviation
SPSS: Statistical Package for Social Sciences
SBP: Systolic blood pressures
TPH: Tryptophan hydroxylase
WC: Waist circumference
WHR: Waist to hip ratio
VDR: Vitamin D receptor

## Footnotes

1 DNA: deoxyribonucleic acid.
